# EchoBack‐CAR T cells: Tuning immunity with sound

**DOI:** 10.1002/ctm2.70391

**Published:** 2025-07-01

**Authors:** Longwei Liu, Yingxiao Wang

**Affiliations:** ^1^ Alfred E. Mann Department of Biomedical Engineering University of Southern California Los Angeles California USA

**Keywords:** car‐t, immunotherapy, solid tumour, ultrasound

1

Despite the transformative success of chimeric antigen receptor T cell (CAR T) therapy in hematologic malignancies, extending this paradigm to solid tumours has proven difficult. Major barriers such as on‐target off‐tumour toxicity, antigen heterogeneity, T cell exhaustion, and poor cell persistence have limited clinical translation in solid tumours.^1^, ^2^ In our recent study, we present EchoBack‐CAR T cells, a novel synthetic biology platform that integrates genetic circuit engineering with focused ultrasound (FUS) control, to address these core limitations with a non‐invasive, spatiotemporally tunable strategy.^3^

This work builds on our team's efforts at the interface of synthetic immunology, T cell biology and physical modulation based on sound. Previously, we developed the first‐generation FUS‐CAR, an ultrasound‐controllable CAR T platform that significantly improved the safety profile of CAR T therapies by spatially confining activation to the tumour site.^4^ However, we also recognized a limitation: the CAR expression in FUS‐CAR T cells was transient and decayed quickly after stimulation which necessary the incorporation of Cre recombinase to enable permeant CAR expression after stimulation. At the same time, our group developed a mammalian cell screening platform for fluorescent biosensor screening and live CAR T cell imaging, which enabled us to dissect endogenous CAR T cell signalling networks with high spatiotemporal resolution.^5^ This insight proved crucial: by mapping the activation dynamics of pathways in CAR T cells engaging tumour antigens, we gained a systems‐level understanding of how to harness natural signalling feedback loops to sustain CAR expression.

The EchoBack CAR design lies the synergy between biophysical control and cellular logic. We first repurposed the high‐throughput evolutionary screening platform for promoter screening to identify a highly heat‐inducible promoter with minimal basal activity. This promoter is activated specifically when cells are exposed to a short pulse of ultrasound and its generated localized heat (e.g., 43°C for 15 min). The resulting EchoBack‐CAR T cells exhibit tightly regulated CAR expression and robustly activated upon ultrasound stimulation at the tumour site. However, achieving durable anti‐tumour activity requires more than a transient pulse of expression. We, therefore, incorporated a positive feedback loop into the genetic design. This circuit senses endogenous T cell signalling pathways activated by tumour engagement, specifically NFAT, NF‐κB, and cAMP/MAPK pathways, and converts those signals into sustained CAR expression. The result is a synthetic immune cell that amplifies its own activation signal, maintaining cytotoxic function long after the initial FUS trigger without continuous external input.

This system overcomes a major drawback of traditional inducible CAR designs, where CAR expression quickly decays and is vulnerable to downregulation upon antigen contact. In both 2D co‐culture and 3D glioblastoma spheroid models, EchoBack‐hGD2CAR T cells demonstrated extended killing efficacy and proliferation compared to standard FUS‐CAR T cells. In vivo, EchoBack‐CAR T cells achieved potent tumour suppression in both subcutaneous and orthotopic glioblastoma models, with improved mouse survival and no off‐tumour toxicity. We further validated this design by applying it to PSMA‐targeted CAR T cells for prostate cancer. Here, EchoBack‐PSMACAR T cells eradicated tumours with no detectable damage to normal tissues expressing low PSMA levels, such as kidney or small intestine while the standard constitutive CAR T cells did. A bilateral tumour model, with a PSMA^low^ distal tumour mimicking healthy tissue, confirmed that EchoBack‐CAR T cells activated by FUS eliminated only the intended target while sparing antigen‐expressing tissues outside the stimulation zone.

Mechanistically, single‐cell RNA sequencing revealed that EchoBack‐CAR T cells adopt a distinct transcriptional profile under chronic antigen exposure. Compared to constitutive CAR T cells, EchoBack cells were enriched in proliferative and cytotoxic CD8+ subsets, with reduced expression of exhaustion markers. Flow cytometry and cytotoxicity assays further confirmed that EchoBack‐CAR T cells resist functional decline after repeated tumour stimulation, likely benefiting from intermittent “rest” periods when not externally activated.

EchoBack‐CAR T cells represent a convergence of synthetic immunology, genetic circuit engineering, and physical modulation. This platform delivers what solid tumour CAR therapy has long lacked: spatial precision, temporal control, and programmable persistence, without sacrificing potency. The EchoBack CAR platform is modular and readily adaptable to diverse antigens, enabling broader targeting without sacrificing safety. In addition to CARs, the same logic could be extended to co‐expressed transgenes, such as checkpoint inhibitors, cytokines, or reprogramming tools.^6^ We envision applications beyond oncology, including autoimmunity, gene editing and regenerative medicine, where remote, reversible gene control is needed.

As tumour cells evolve under therapeutic pressure, future engineering must address challenges such as antigen escape and the immunosuppressive tumour microenvironment. Priorities include reprogramming T cells to overcome local inhibition, refining ultrasound delivery to reduce thermal impact, and implementing closed‐loop systems for real‐time, feedback‐controlled stimulation. These innovations will be critical to enhancing the safety, precision and robustness of EchoBack CAR‐T cell therapies. Looking ahead, we envision ultrasound‐guided cellular therapies becoming a core platform in the next generation of precision immunotherapy.
